# Direct anterior approach versus posterolateral approach for total hip arthroplasty in the treatment of femoral neck fractures in elderly patients: a meta-analysis and systematic review

**DOI:** 10.1080/07853890.2023.2193424

**Published:** 2023-03-31

**Authors:** Zhiqiang Jin, Lingge Wang, Jun Qin, Hao Hu, Qingjun Wei

**Affiliations:** a Department of Orthopedic Trauma and Hand Surgery, The First Affiliated Hospital of Guangxi Medical University, Nanning, P. R. China; b Department of Orthopedics, The Affiliated Zhongshan Hospital of Dalian University, Dalian, Liaoning, P. R. China; c Department of Spinal Surgery, The First Affiliated Hospital of Guangxi Medical University, Nanning, P. R. China

**Keywords:** Direct anterior approach, posterolateral approach, total hip arthroplasty, meta-analysis

## Abstract

**Objective:**

The purpose of this meta-analysis was to evaluate the postoperative clinical outcomes of elderly patients who underwent the direct anterior approach (DAA) versus those who received posterolateral approach (PLA) for total hip arthroplasty (THA) in the treatment of femoral neck fractures.

**Methods:**

An electronic search was conducted in databases including PubMed, Embase, Web of Science, the Cochrane Library, and CNKI from their inception to January 2022. We calculated the odds ratio (OR) and mean difference (MD) with 95% confidence intervals (CIs) to assess the effect of DAA compared to PLA for the management of total hip arthroplasty (THA) in elderly patients using the dichotomous or continuous method with a random or fixed-effect model.

**Results:**

15 studies involving 1284 patients were included; 640 patients receiving DAA and 644 patients receiving PLA. DAA had a longer surgery duration than PLA [WMD = 9.41, 95% CI (4.64, 14.19), *I*^2^=95.5%]; The amount of postoperative drainage [WMD= −3.88, 95% CI (−5.59, −2.17), *I*^2^=98.3%], length of incision [WMD= −3.88, 95% CI (−5.59, −2.17), *I*^2^=98.3%], blood loss [WMD= −3.88, 95% CI (−5.59, −2.17), *I*^2^=98.3%], hospitalization time [WMD= −3.88, 95% CI (−5.59, −2.17), *I^2^*=98.3%], and postoperative bedtime [WMD = −5.56,95% CI (−7.11, −4.01), *I*^2^=99.0%], were similar between the two groups (*p* < 0.05). The HHS at 1 month, 12 months postoperatively [WMD = 7.58, 95%CI (5.70,9.46), *I*^2^=89.5%; WMD= 2.56, 95%CI 0.11,5.00, *I*^2^=93.2%] and the incidence of LFCN in patients were higher in the DAA group (OR = 2.91, 95% CI 1.26 to 6.71, *I*^2^=0.0%), while fewer patients in the DAA group suffered from postoperative dislocation than in the PLA group (OR = 0.26, 95% CI 0.11 to 0.60, *I*^2^=0.0%). No significant difference was observed in HHS at 1 week, 3 months, and 6 months postoperatively, VAS postoperatively at each time point, acetabular anteversion angle, acetabular abduction angle, wound infection, deep vein thrombosis, and intraoperative fracture (*p* > 0.05).

**Conclusions:**

DAA offers a quicker functional recovery and is less invasive with an earlier return to daily activities in older THA patients than PLA. However, DAA was found to be associated with a high incidence of lateral femoral cutaneous nerve injury and a low incidence of postoperative dislocation.

**Key messages**
- The present study aims to evaluate the clinical outcomes in elderly patients receiving DAA versus PLA for THA in the treatment of femoral neck fractures by mate-analysis.
- DAA offers a quicker functional recovery and is less invasive with an earlier return to daily activities in older THA patients. No significant difference was observed between the colchicine and comparators in terms of the need for HHS at 1 week, 3 months, and 6 months postoperatively, VAS postoperatively, acetabular anteversion angle, acetabular abduction angle, and complications (wound infection, deep vein thrombosis, and intraoperative fracture).

## Introduction

The treatment of patients suffering from hip problems aims to help patients recover from normal hip activities and functions [1]. The hip replacement surgery performed on patients is affected by patients’ geographic area, socio-economic status, gender, race, and age [2,3]. Total hip arthroplasty (THA) increases the risk of health hazards in older patients, given their deteriorating health conditions [4]. An appropriate surgery technique for THA can relieve pain, improve hip function, and shorten hospital stay and recovery time thus gaining patients’ satisfaction [5,6]. Generally, surgeons should weigh up the pros and cons before performing THA on elder patients with multimorbidity. Nevertheless, no precise guidelines are available to surgeons on selecting the most desirable approach. THA can be performed using a variety of surgical approaches, such as the direct anterior approach (DAA), the anterolateral approach, the lateral approach, and the posterolateral approach (PLA) [7–9]. Several reviews have investigated different surgical methods for THA in patients of all ages [10–14]. According to Elina Huerfano et al. [15], there was no statistically significant difference in the incidence of dislocation related to surgical factors or cup positioning between the DAA and PLA groups. As a result, after primary THA, surgical techniques had minimal impact on prosthesis instability. Elina Huerfano et al. focused on prosthesis instability after primary THA for all ages, while we are more concerned about intraoperative and postoperative clinical investigations only for elder patients. Zhang Jiakai et al. [16] compared the effects of DAA and other methods on patients regarding femoral neck fractures. They found that DAA for hemiarthroplasty was associated with decreased dislocation rate in comparison with the posterior capsular approach. However, the effects of DAA versus PLA for THA in elderly patients with hip problems were not investigated, as the methods used to be compared with DAA in their study did not include PLA, and only patients with femoral neck fractures were investigated. Sun Xuedong et al. [17] compared DAA and PLA through a network meta-analysis and they discovered higher HHS in 6-month postoperative periods and shorter hospital stay in the DAA group compared with the PLA group, suggesting that DAA was more effective than the PLA regarding the postoperative early recovery of function. However, their study did not investigate the effects of DAA and PLA only on elder patients. Hence, the present study aims to evaluate the clinical outcomes in elder patients receiving DAA versus PLA for THA by meta-analysis.

## Methods

### Search strategy and selection criteria

This meta-analysis was conducted in accordance with the Preferred Reporting Items for Systematic Reviews and Meta-Analyses (PRISMA) Statement. And the protocol was registered with the International Prospective Register of Systematic Reviews [18].

### Search strategy

The following databases were searched from their inception to January 2022, including Embase, PubMed, Cochrane library, Web of Science, and CNKI, with no restrictions on language. The following combined text and Medical Subject Headings (MeSH) terms were used: (Arthroplasty, Replacement, Hip[Mesh]); total hip replacement; total hip arthroplasty; direct anterior approach, posterolateral approach, THA, THR, DAA, and PLA, Aged[Mesh] OR Aged[Title/Abstract] OR Elderly[Title/Abstract]) OR old people[Title/Abstract]) OR elder[Title/Abstract]) OR Geriatric[Title/Abstract]. The search used for PubMed was: (Arthroplasty, Replacement, Hip[Mesh] OR Arthroplasty, Replacement[Title/Abstract] OR Arthroplasties, Replacement, Hip[Title/Abstract] OR Arthroplasty, Hip Replacement[Title/Abstract]) OR Hip Prosthesis Implantation[Title/Abstract]) OR Hip Prosthesis Implantations[Title/Abstract]) AND Direct anterior[Title/Abstract]) AND posterolateral[Title/Abstract] AND (Aged[Mesh] OR Aged[Title/Abstract] OR Elderly[Title/Abstract]) OR old people[Title/Abstract]) OR elder[Title/Abstract]) OR Geriatric[Title/Abstract]). All studies potentially eligible for review were considered, regardless of primary outcome or language.

### Literature selection and data extraction

Studies were included in this meta-analysis if they were randomized clinical trials (RCTs) or controlled clinical trials which compared DAA and PLA for THA in elderly patients with a femoral neck fracture. We included elderly patients at age of over 60. We excluded studies if their full texts were not available, or their original research cannot be extracted. Excluded study types included animal experiments (such as pharmacological or pharmacokinetic studies), case reports, abstracts, editorial comments, conference papers, reviews, meta-analyses, etc. Duplicate publications were also excluded. The titles and abstracts were screened by two investigators independently to identify studies that met the inclusion criteria for full-text assessment. Two investigators analysed the selected studies and extracted data with an agreement value (κ) of 96·5%. Any disagreements were resolved by a third investigator. The extracted data included the name of the first author, publication year, country, general characteristics of patients (age, proportion of female patients, and BMI), outcomes, study and follow-up duration. The primary outcomes were Harris hip score (HHS) at 1 week, 1 month, 3 months, 6 months, and 12 months postoperatively; VAS at 3 days, 7 days, 1 month, and 6 months postoperatively; length of incision, duration of surgery, blood loss, length of hospital stay, postoperative drainage, acetabular abduction angle, acetabular anteversion angle, and complications (intraoperative fracture, postoperative dislocation, lateral femoral cutaneous nerve (LFCN) neurapraxia, wound infection, and deep vein thrombosis). Reference lists of relevant systematic reviews and identified articles also were reviewed to find additional eligible studies as completely as possible. No restriction was placed on the language or country of publication.

### Statistical analysis

Continuous outcomes (Harris hip score at 1 week, 1 month, 3 months, 6 months, and 12 months postoperatively; VAS at 3 days, 7 days, 1 month, 6 months, and 12 months postoperatively; length of incision, duration of surgery, blood loss, length of hospital stay, postoperative drainage, duration of postoperative bed rest, acetabular abduction angle, acetabular anteversion angle) were reported as weighted mean differences (WMD) with 95% confidence intervals (CIs). As for the meta-analysis of dichotomous outcomes (intraoperative fracture, postoperative dislocation, lateral femoral cutaneous nerve (LFCN) neurapraxia, wound infection, and deep vein thrombosis), the odds ratio (OR) with 95% CIs was used. *p* < 0.05 was considered the threshold of significant difference. The funnel plot was adopted to assess publication bias (as shown in the Supplement) for the effect size against the standard error in each eligible study. Begg’s test was performed to examine the asymmetry of the funnel plot. with *p* < 0.05 suggesting publication bias [19]. We performed the Chi-square test to evaluate heterogeneity between studies [20] and the I^2^ test to assess the magnitude of the heterogeneity, with *I*^2^ value over 50% indicating high heterogeneity [21]. Software Stata version 15.1 (Stata Corp, College Station, TX, USA) was utilized for all statistical analyses.

### Funding source

This study was funded entirely by intramural funds without the involvement of any commercial entities. The funding source was not involved in the design of the study, data collection, data analysis, data interpretation, or report writing. The corresponding author had full access to all study data and was ultimately responsible for the decision to submit it for publication.

## Results

### Eligible studies and their characteristics

**Figure 1.**
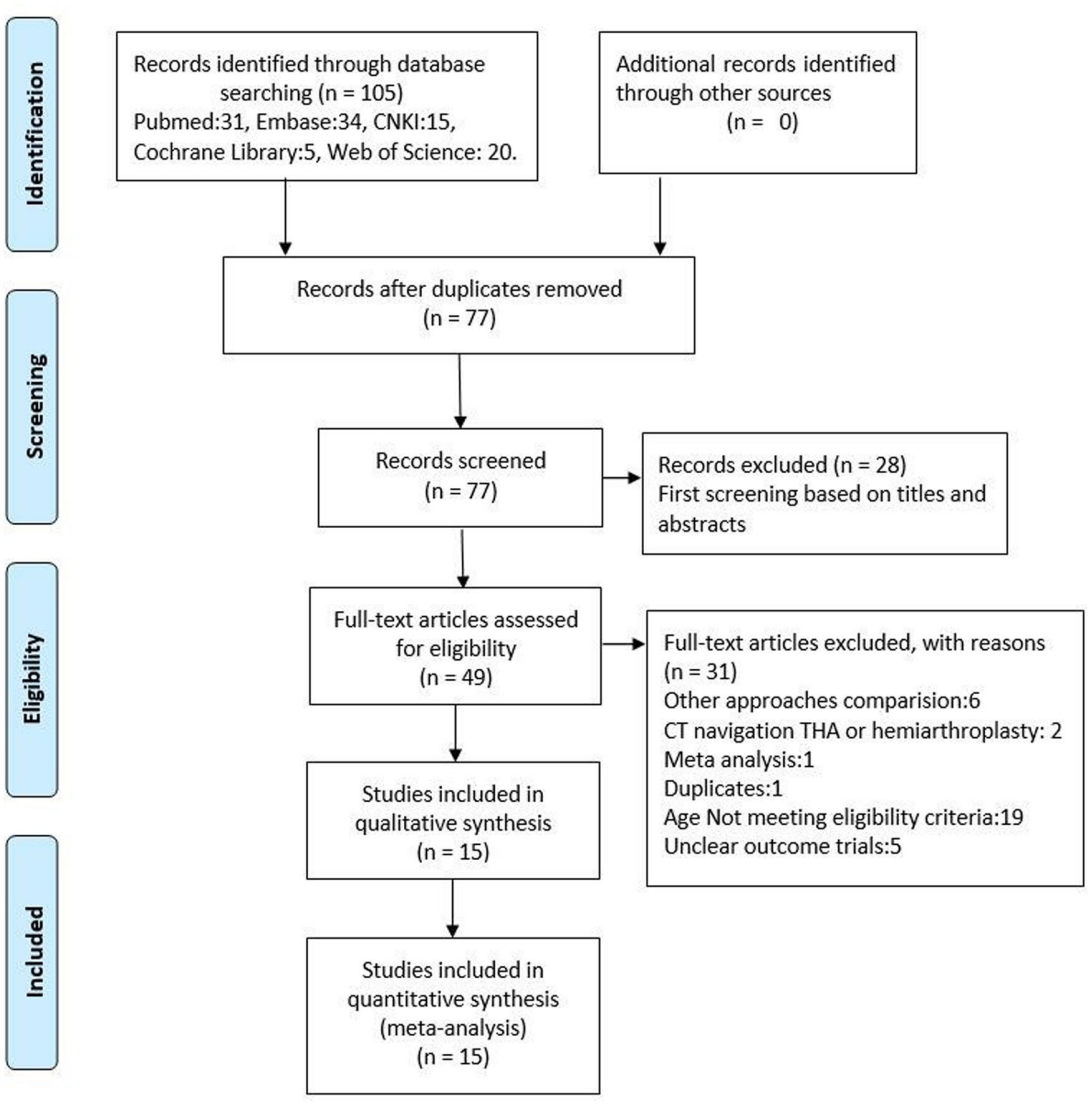
The flow diagram of study selection.

The literature retrieval and selection process is presented in Figure 1. A total of 105 studies were initially identified from databases (Web of Science = 20, Embase = 34, PubMed = 31, Cochrane Library = 5, CNKI =15). Endnote X8 (Thomson Reuters Corp, USA) software was utilized to remove duplicate publications, with 77 studies left. After screening the titles and abstracts, 28 studies were removed. Finally, 15 studies with 1,284 patients (DAA group = 640, PLA group = 644) that met the inclusion criteria were included in the meta-analysis [22–36], and the basic characteristics of included studies are summarized in Table 1. The publication year was from 2015 to 2021. Follow-up duration ranged from 1 week to 1 year.

**Table 1.** General characteristics of included studies.

Authors	Years	Study design	Sample size (D/P)	Approaches	Age (Mean ± SD)	Gender (F/M)	BMI (kg/m^2^)	Follow up	Outcome
Denglu Yan	2015	Retrospective study	71(37/34)	DAA	71.47 ± 8.34	21/16	NS	24 months	11,12,13,16,20
				PLA	72.13 ± 9.48	16/18	NS		
He Yong	2017	Retrospective study	60(30/30)	DAA	76.5 ± 6.2	18/12	NS	6 months	1,2,3,4,7,8,9,12,13,14,15,16,20
				PLA	74.2 ± 5.4	14/16	NS		
Wang Yumin	2017	Retrospective study	29(12/17)	DAA	78.70 ± 3.60	7/5	NS	6 months	4,11,12,13,15
				PLA	NS	NS	NS		
Tao Tao	2018	Retrospective study	80(35/45)	DAA	71.55 ± 8.89	25/10	20.5	6 months	2,4,6,11,12,15, 16,17,19,20
				PLA	72.40 ± 8.52	31/14	21.3		
You Sen	2018	Retrospective study	60(30/30)	DAA	71.9 ± 9.2	18/12	22.4	6 months	1,2,3,6,7,11,12,13,14,16,17,18,19,20,21,22
				PLA	76.8 ± 14.3	20/10	23.7		
Ding Shaolong	2019	Retrospective study	82(41/41)	DAA	70.23 ± 5.03	19/22	NS	6 months	11,12,13,14,15,16,20,22,23
				PLA	70.67 ± 4.83	16/25	NS		
Cheng Rui	2020	Retrospective study	100(50/50)	DAA	69.61 ± 3.53	24/26	NS	3 months	1,3,4,6,7,11,12,13,14,16
				PLA	70.48 ± 4.21	23/27	NS		
Li Rui	2020	Retrospective study	68(34/34)	DAA	75.23 ± 11.45	15/19	NS	12 months	8,9,11,12,13,15,17,18,19,20,22,23
				PLA	74.73 ± 12.67	16/18	NS		
He Guo	2021	Retrospective study	132(68/64)	DAA	69.05 ± 3.27	39/29	NS	12 months	2,4,5,8,9,11,12,13,16,17,18,20,21,22
				PLA	68.95 ± 3.17	35/29	NS		
Li Jinguang	2021	Retrospective study	84(42/42)	DAA	64.33 ± 2.35	21/21	NS	6 months	2,4,11,12,13,14,20,21
				PLA	65.61 ± 2.33	20/22	NS		
Li Junran	2021	Retrospective study	171(89/82)	DAA	68.63 ± 4.35	48/41	24.66	12 months	1,2,3,4,5,12,13,21
				PLA	67.93 ± 3.49	46/36	25.22		
Ma Chao	2021	Prospective study	96(48/48)	DAA	73.4 ± 7.6	23/25	24.71	12 months	2,4,5,7,11,12,13,14,17,18,20,21,23
				PLA	73.1 ± 7.5	21/27	24.52		
Sun Yuexian	2021	Retrospective study	84(42/42)	DAA	71.02 ± 4.86	24/18	NS	3 months	3,12,13,15,16, 20,22,23
				PLA	70.75 ± 5.34	25/17	NS		
Wang Yongcai	2021	Retrospective study	60(30/30)	DAA	NS	13/17	NS	6 months	2,3,4,12,13,21, 22
				PLA	NS	14/16	NS		
Zhang Xiaomin	2021	Retrospective study	107(52/55)	DAA	70.15 ± 6.16	34/18	22.37	12 months	1,2,3,5,6,7,8,11,12,13
				PLA	70.02 ± 5.86	35/20	22.82		

NS: not stated. (1) Harris hip score at 1 week, (2) Harris hip score at 1 month, (3) Harris hip score at 3 months, (4) Harris hip score at 6 months, (5) Harris hip score at 12 months; (6) VAS at 3 days, (7) VAS at 7 days, 8. VAS at 1 month, (9) VAS at 6 months, (10) VAS at 12 months, (11) skin incision length, (12) operation time, (13) blood loss, (14) hospitalization time, (15) postoperative drainage, (16) duration of postoperative bed rest, (17) acetabular abduction angle, (18) acetabular anteversion angle, (19) intraoperative fracture, (20) postoperative dislocation, (21) lateral femoral cutaneous nerve (LFCN) neurapraxia, (22) wound infection, (23) deep vein thrombosis.

### Risk of bias

**Figure 2.**
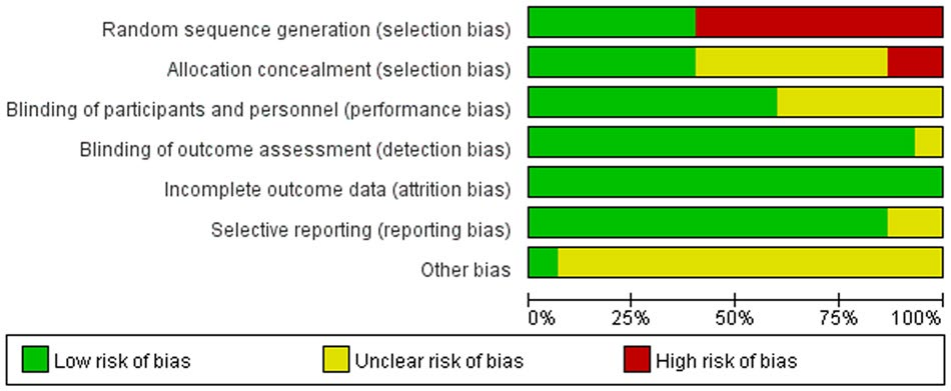
Risk of bias graph: review authors’ judgments about each risk of bias item presented as percentages across all included studies.

**Figure 3.**
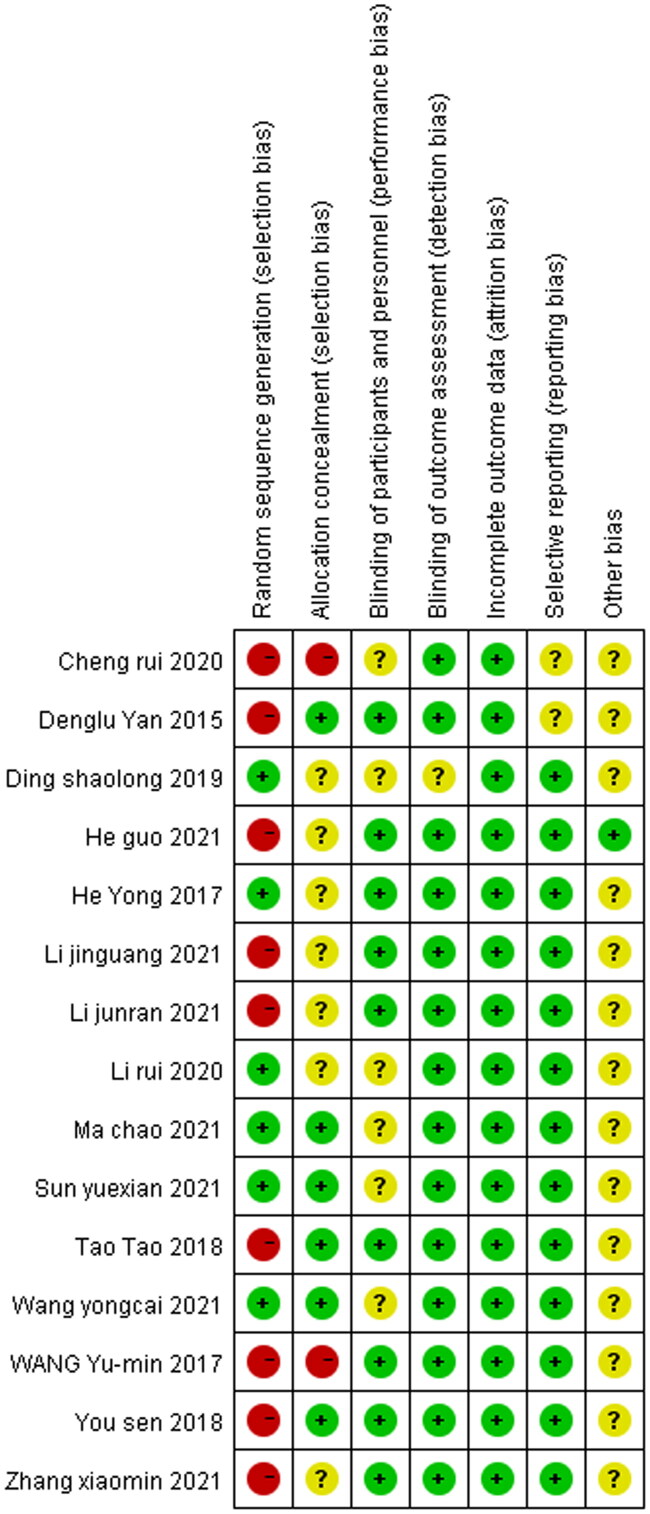
Risk of bias summary for included studies. +, no bias; −, bias; ?, bias unknown.

The risk of bias graph and summary across included studies are presented in Figures 2 and 3 respectively. Among 15 eligible studies, 2 were rated with a low risk of bias (selection bias), and 5 were judged with unclear risk of bias (selection bias). The risk of bias about allocation concealment was low in 4 studies but high in the other 2 studies. The risk of bias about the blinding of participants was high across all eligible studies. The risk of bias regarding attrition was unclear in 6 studies. The risk of bias from other sources was high in 1 study and unclear in 2 studies.

### Meta-analysis

#### Harris hip score at 1 week, 1 month, 3 months, 6 months, and 12 months postoperatively

**Figure 4.**
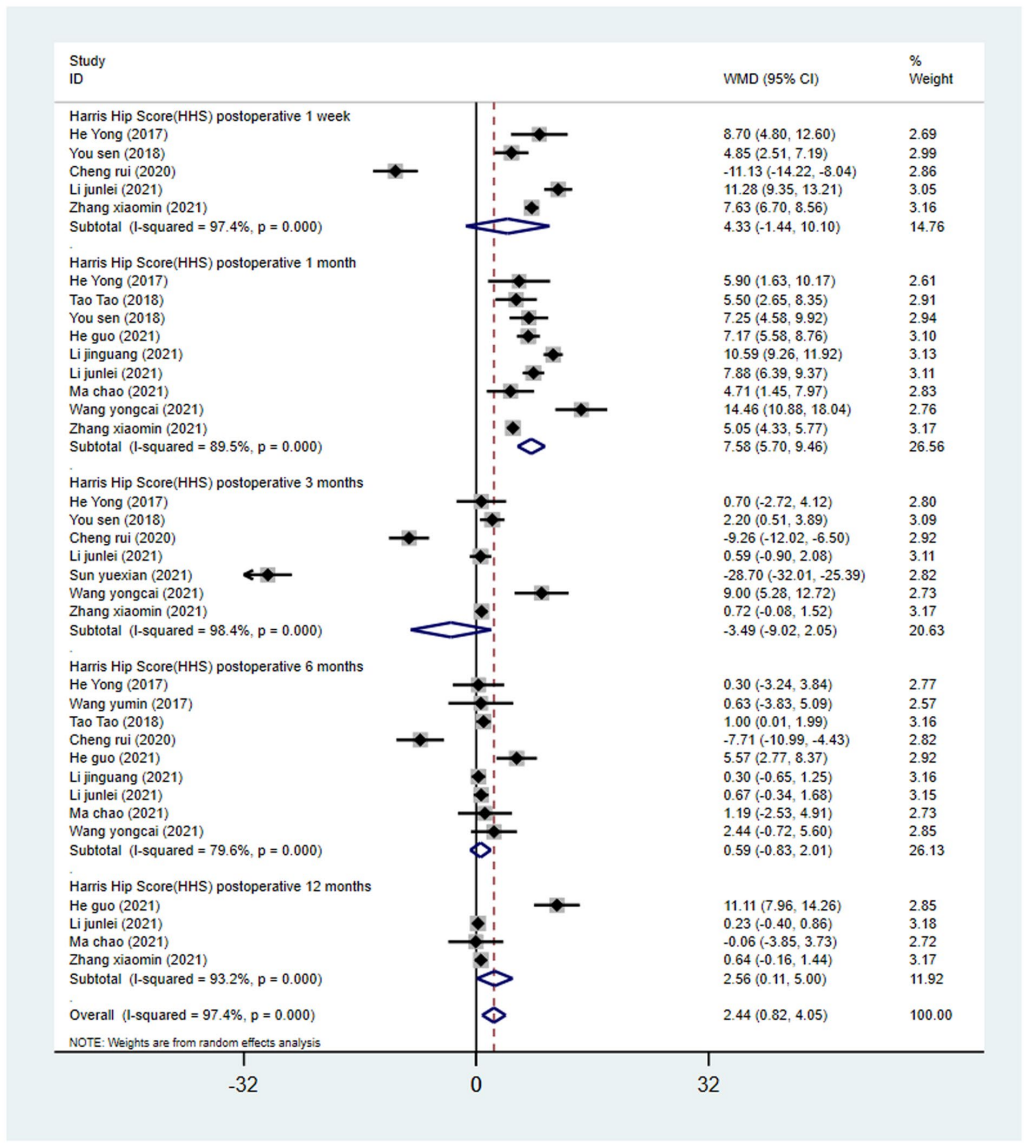
Forest plot for comparing DAA versus PLA in terms of Harris hip score postoperative at 1 week, 1 month, 3 months, 6 months and 12 months.

Among the 15 studies, 5 studies [23,26,28,32,36] reported HHS at 1 week postoperatively, 9 studies [23,25,26,30–33,35,36] reported HHS at 1 month postoperatively, 7 studies [23,26,28,32,34–36] reported HHS at 3 months postoperatively, 9 studies [23–25,28,30–33,35] reported HHS at 6 months postoperatively, and 4 studies [20,28,29,32] reported HHS at 12 months postoperatively. A total of 1,284 patients who received THA were involved in 15 eligible studies (640 in the DAA group and 644 in the PLA group). We conducted the meta-analysis in subgroups based on different follow-up time points. The results showed that HHS at 1 month and 12 months postoperatively were higher in the DAA group than that in the PLA group (1 month, WMD = 7.58, 95%CI 5.70 to 9.46, *p* = 0.000, *I*^2^=89.5%; 12 months, WMD= 2.56, 95%CI 0.11 to 5.00, *p* = 0.040, *I*^2^=93.2%; Figure 4). No statistically significant difference was observed between the two surgical approaches in HHS at 1 week, 3 months, and 6 months postoperatively (1 week, WMD = 4.33, 95%CI − 1.44 to 10.10, *p* = 0.142, *I*^2^=97.4%; 3 months, WMD = −3.49, 95%CI −9.02 to 2.05, *p* = 0.217, *I*^2^=98.4%; 6 months, WMD = 0.59, 95%CI −0.83 to 2.01, *p* = 0.415, *I*^2^=79.6%; Figure 4).

#### Visual analogue scale at 3 days, 7 days, 1 month, 6 months, and 12 months postoperatively

**Figure 5.**
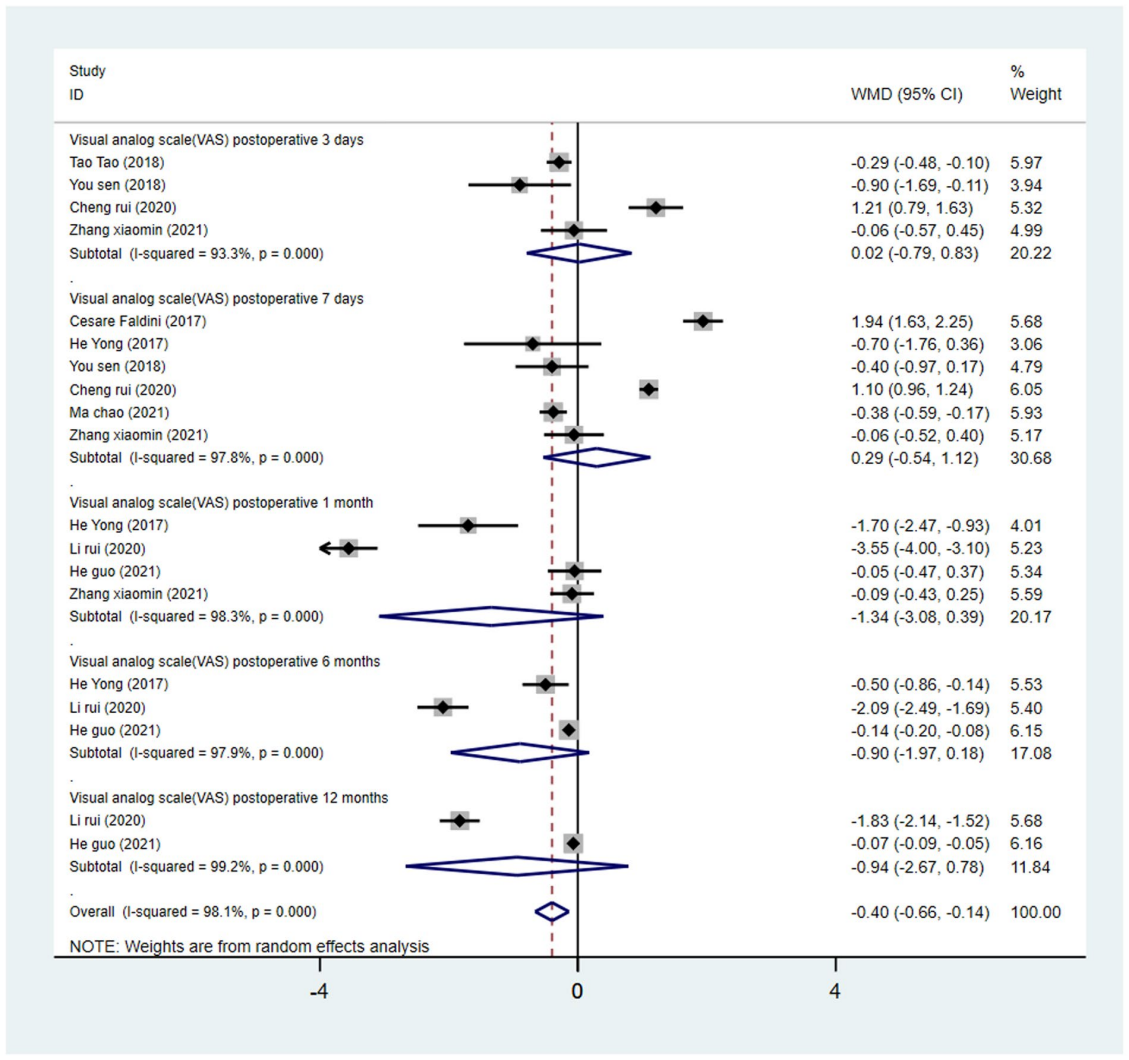
Forest plot for comparing DAA versus PLA in terms of Visual Analogue Scale at 3 days, 7 days, 1 month, 6 months, and 12 months.

Compared with the PLA group, the DAA group was not associated with the VAS at each time point postoperatively in comparison with the PLA group (3 days, WMD = 0.02, 95%CI −0.79 to 0.83, *p* = 0.96; 7 days, WMD = 0.05, 95%CI −0.918 to 0.817, *p* = 0.909; 1 month, WMD = −1.34, 95%CI −3.08 to −0.39, *p* = 0.130; 6 months, WMD = −0.90, 95%CI −1.97 to −0.18, *p* = 0.101; 12 months, WMD = −0.94, 95%CI −0.56 to −0.02, *p* = 0.284; Figure 5).

#### Length of incision

**Figure 6.**
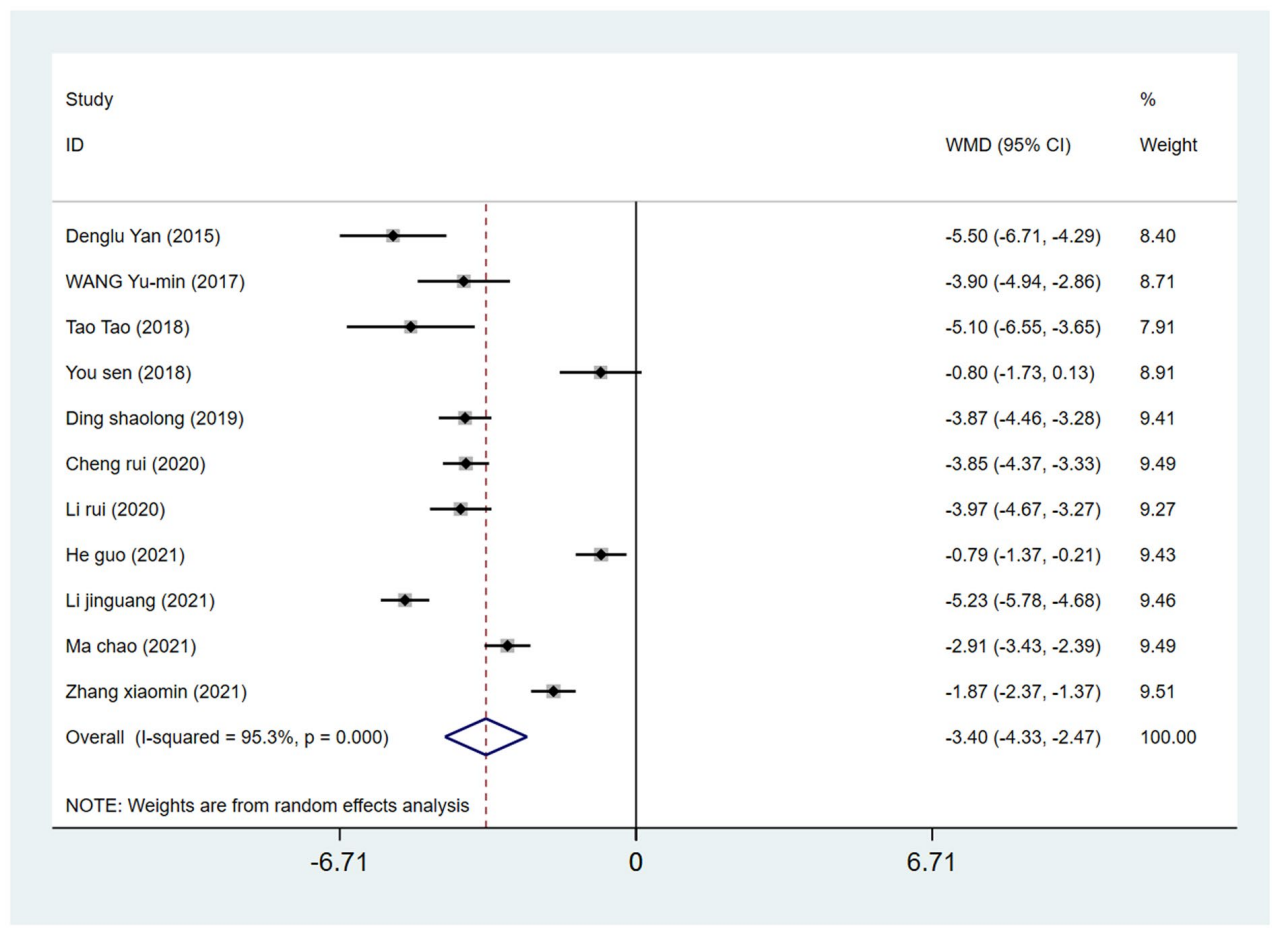
Forest plot for comparing DAA versus PLA in terms of skin incision length.

Eleven studies reported incision length involving 909 patients undergoing THA (449 in the DAA group and 460 in the PLA group). By pooling the data on the length of incision from these studies, the incision length in the DAA group was found to be 3.40 cm shorter than that in the PLA group. (WMD = −3.40, 95%CI −4.33 to −2.47, *I*^2^=95.3%, *p* = 0.000, Figure 6).

#### Duration of surgery

**Figure 7.**
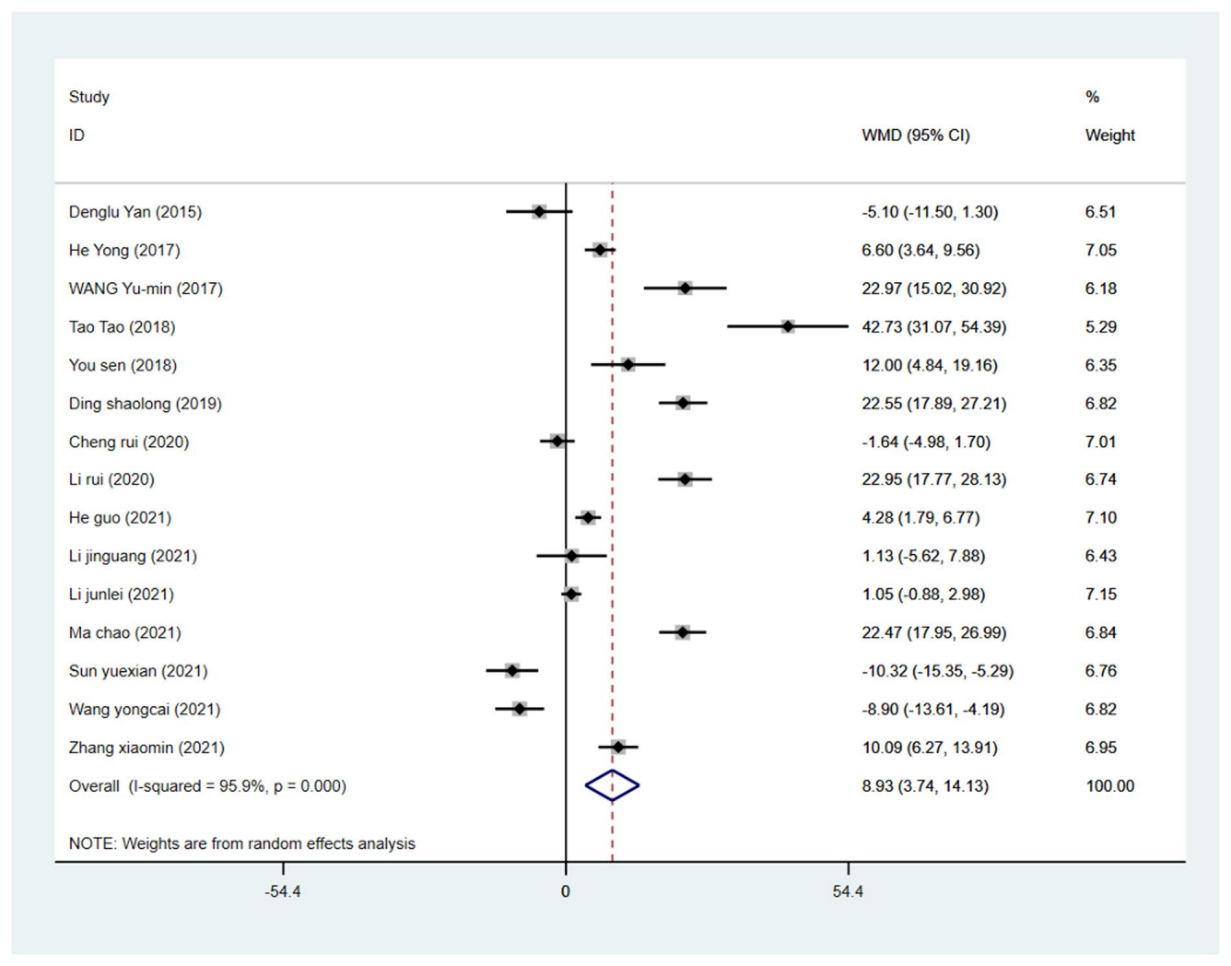
Forest plot for comparing DAA versus PLA in terms of operation time.

The duration of surgery was reported in all 15 included studies involving 1284 patients (640 in the DAA group and 644 in the PLA group). We pooled the data on surgery duration from these studies and found that compared with the PLA group, the increase in the surgery duration was not related to the DAA approach (WMD= 8.93, 95%CI 3.74 to 14.127, *p* = 0.001, *I*^2^=95.9% Figure 7).

#### Blood loss

**Figure 8.**
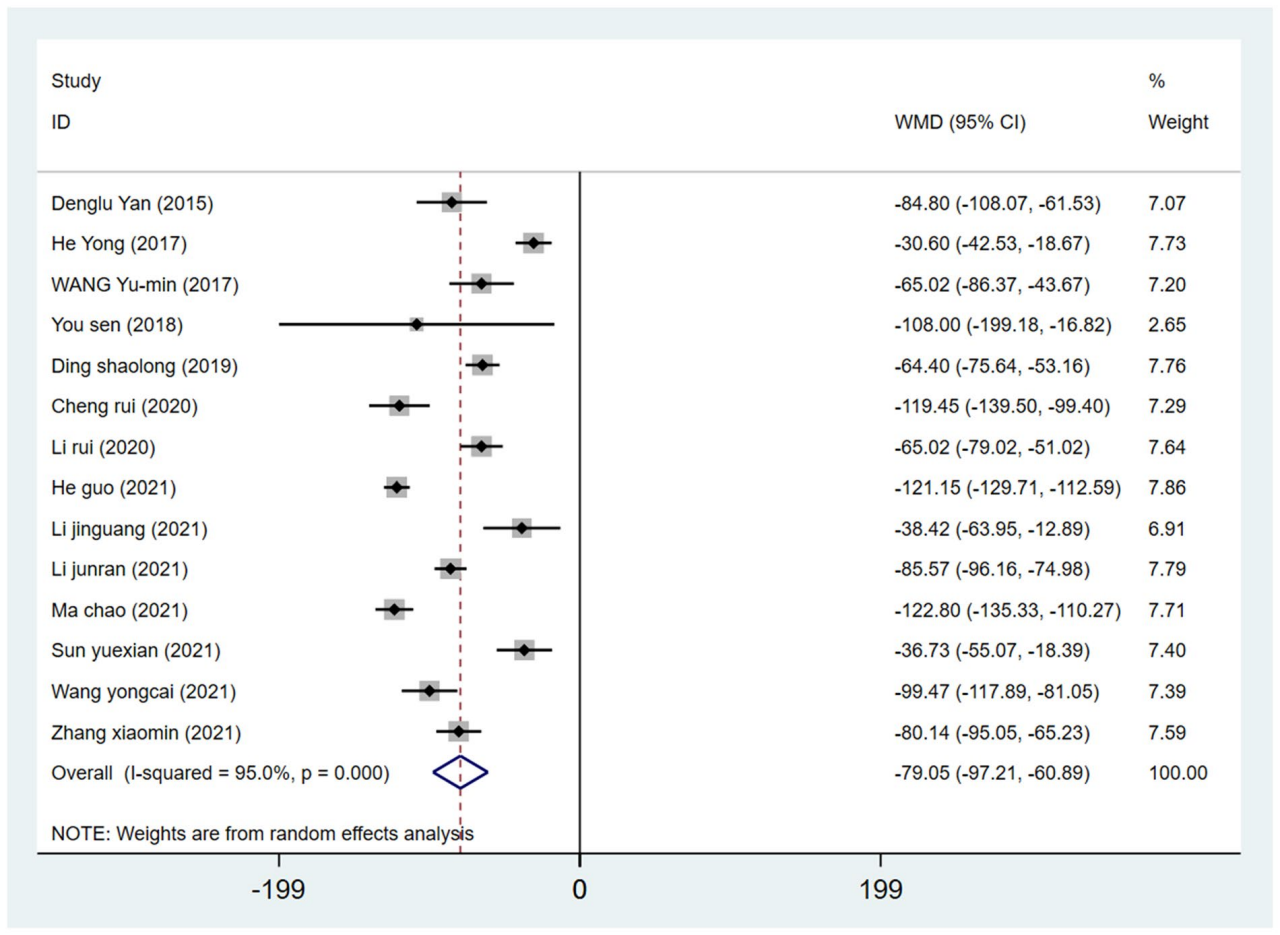
Forest plot for comparing DAA versus PLA in terms of blood loss.

Fourteen studies reported blood loss involving 1204 patients (605 in the DAA group and 599 in the PLA group). We pooled the data on blood loss from these studies and discovered that the postoperative blood loss in the DAA group was less than that in the PLA group (WMD = −79.05, 95%CI −97.21 to −60.89, *I*^2^=95.0%, *p* = 0.000, Figure 8).

#### Length of stay in hospital

**Figure 9.**
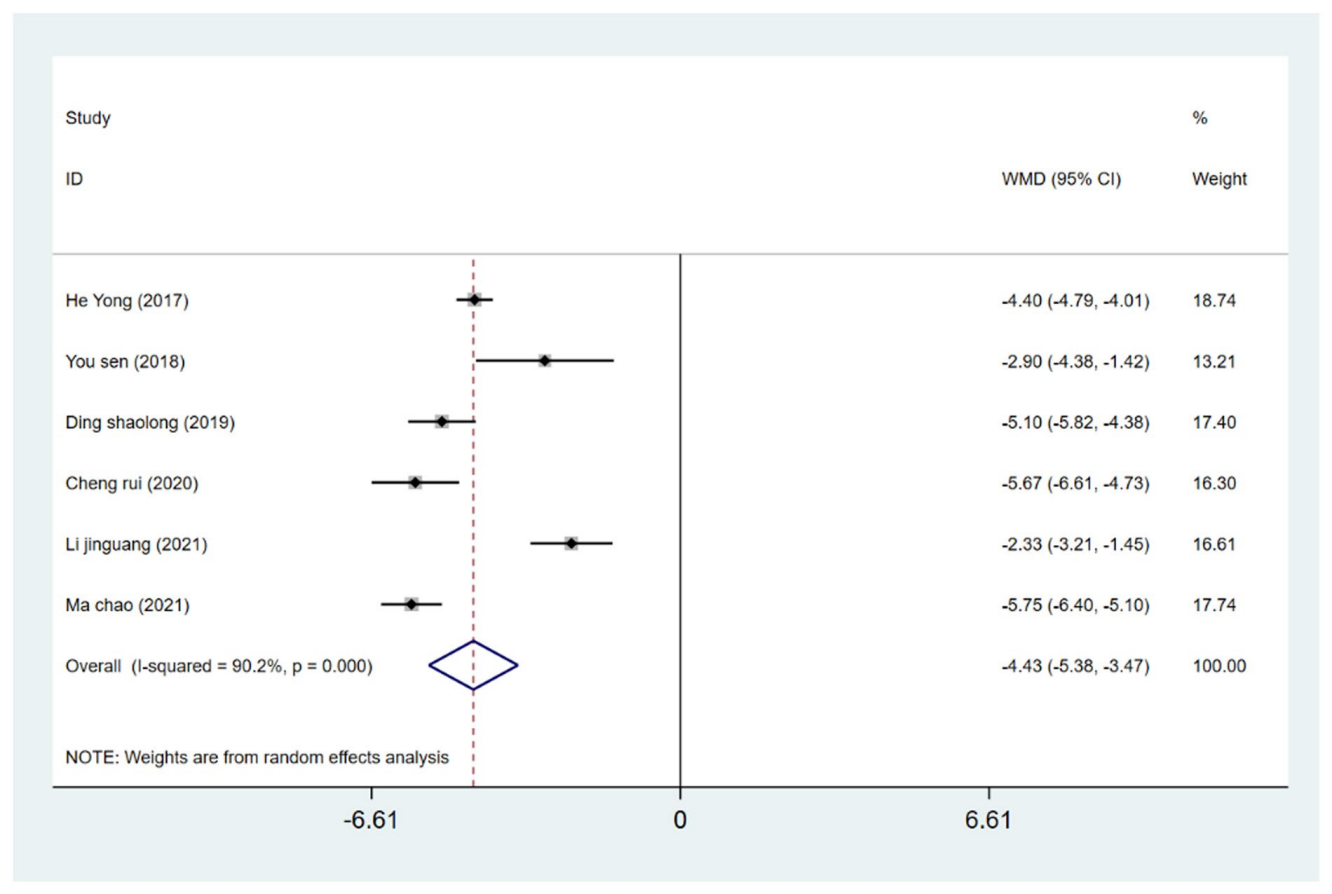
Forest plot for comparing DAA versus PLA in terms of hospitalization time.

The length of hospital stay was reported in six studies involving 482 patients (241 in the DAA group and 241 in the PLA group). A random-effects model was utilized for meta-analysis due to significant heterogeneity across eligible studies (*I*^2^ > 50%) which could not be eliminated by either sensitivity analysis or subgroup analysis. The results revealed no significant difference between the two groups regarding the hospital length of stay (WMD= −4.43, 95%CI −5.38 to −3.47, *p* = 0.000, Figure 9).

#### Postoperative bed rest duration

**Figure 10.**
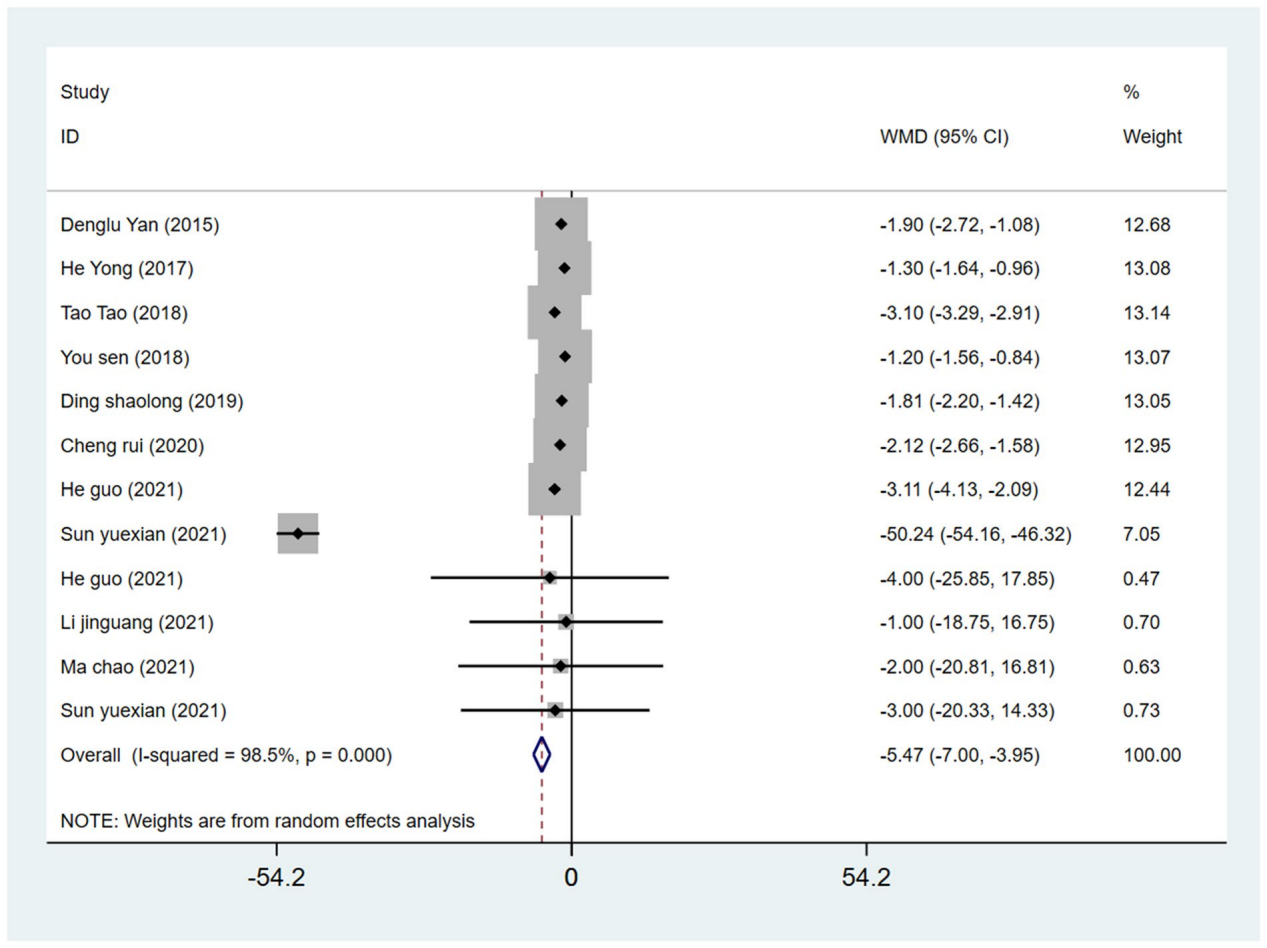
Forest plot for comparing DAA versus PLA in terms of the duration of postoperative bed rest.

The WMD of the duration of postoperative bed rest for the DAA group which was −5.47 (*p* = 0.000; 95%CI −7.00 to −3.95), was lower than that for the PLA group, with a significant difference between the two groups (*p* < 0.05) (Figure 10).

#### Acetabular abduction angle

**Figure 11.**
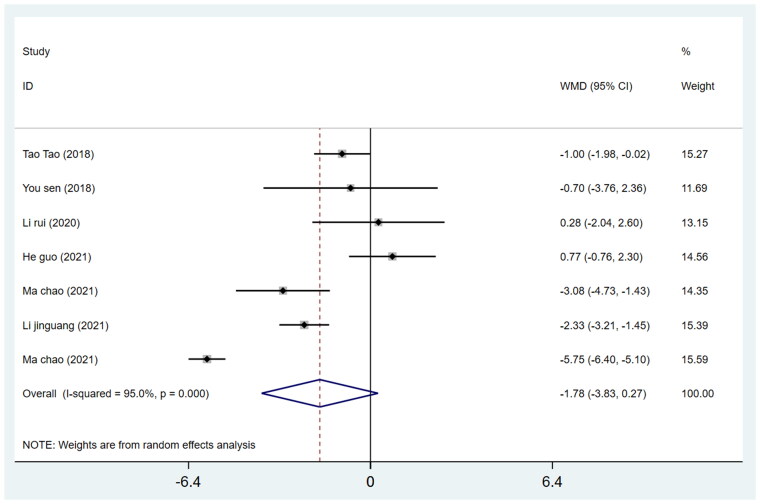
Forest plot for comparing DAA versus PLA in terms of acetabular abduction angle.

We pooled data from 7 studies on 436 patients (215 in the DAA group and 221 in the PLA group) regarding the acetabular abduction angle of THA. The WMD of the inclination angle through DAA was −1.78 (*p* = 0.088; *I*^2^=95.0%,95%CI − 3.83 to −0.27), while between the two approaches, there was no significant difference in the acetabular abduction angle (*p* > 0.05) (Figure 11).

#### Acetabular anteversion angle

**Figure 12.**
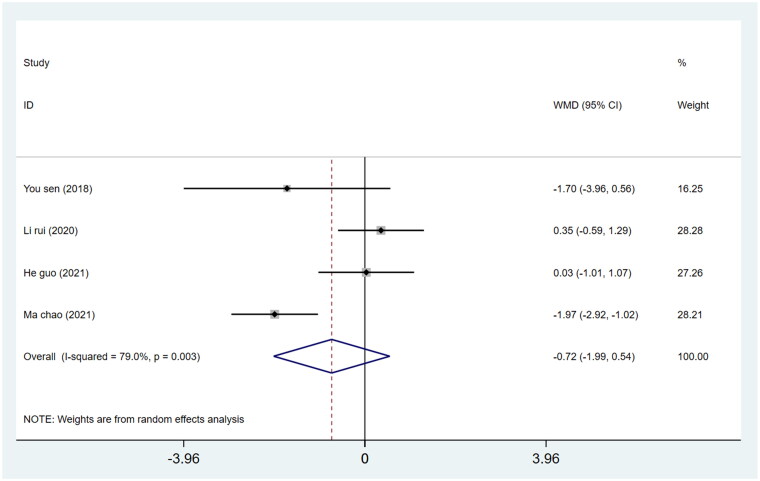
Forest plot for comparing DAA versus PLA in terms of acetabular anteversion angle.

We pooled data on 356 patients (180 in the DAA group and 176 in the PLA group) from 7 studies regarding the acetabular abduction angle of THA. For the DAA method, the WMD of the anteversion angle was −0.72 (*p* = 0.263; 95%CI −1.99 to 0.54), with no significant difference in the acetabular abduction angle between the two groups (*p* > 0.05) (Figure 12).

#### Complications

**Figure 13.**
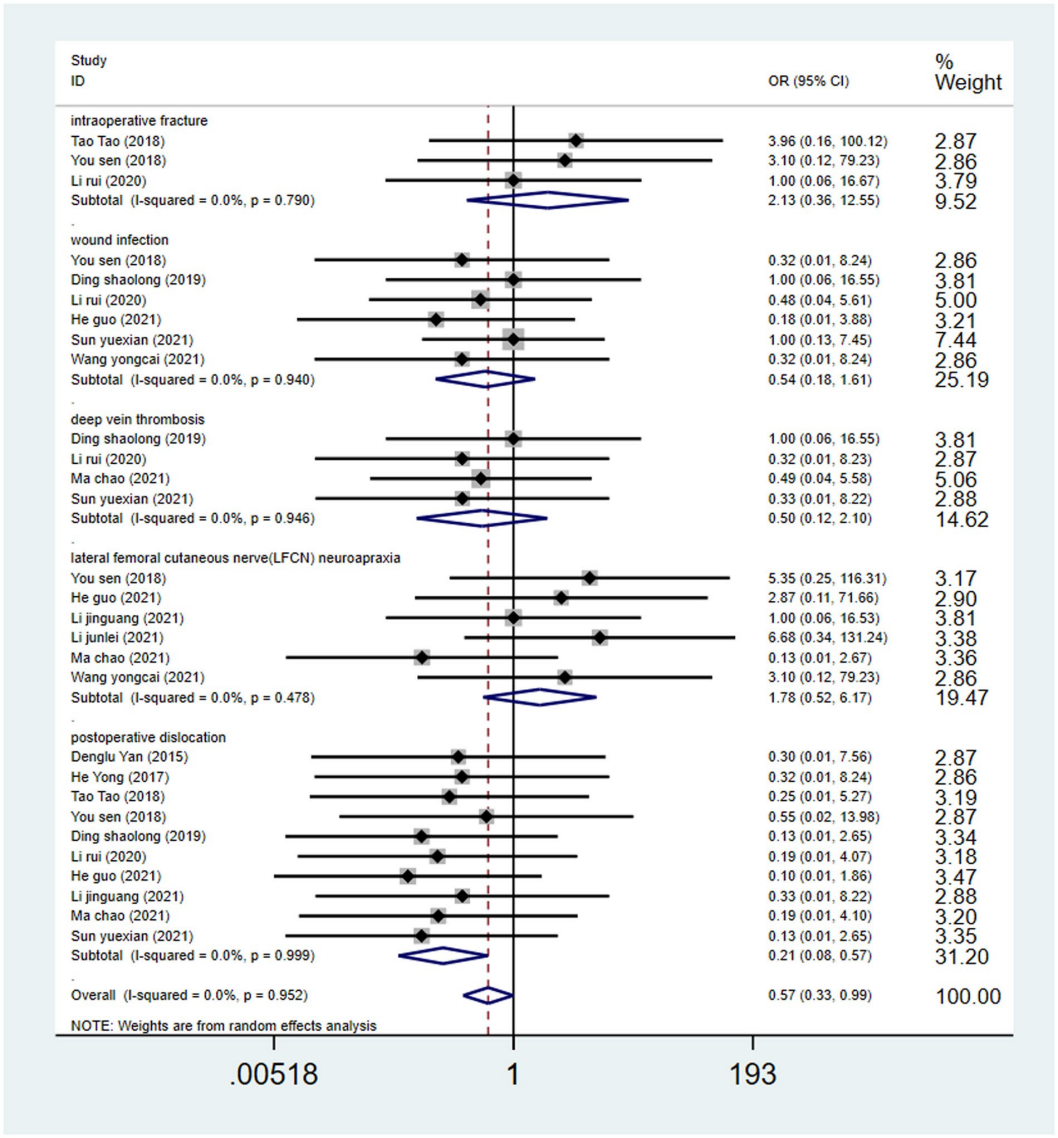
Forest plot for comparing DAA versus PLA in terms of complications.

No significant difference emerged between the DAA and the PLA group in terms of intraoperative fracture (OR = 2.20, 95%CI 0.398 to 12.191, *p* = 0.366, Figure 13), wound infection (OR = 0.51, 95%CI 0.18 to 1.45, *p* = 0.204, Figure 13), and deep vein thrombosis (OR = 0.49, 95%CI 0.12 to 2.00, *p* = 0.320, Figure 13). During the follow-up period, the incidence of the lateral femoral cutaneous nerve (LFCN) neurapraxia was found to be higher in the DAA group than that in the PLA group (OR = 1.78; 95%CI 0.52 to 6.17, *p* = 0.358, Figure 13). However, complete recovery was recorded respectively at 3- and 6-month follow­up. Moreover, The DAA group was also related to a higher incidence of postoperative dislocation compared with the PLA group (OR = 0.201; 95%CI 0.08 to 0.53, *p* = 0.001, Figure 13) [17,33,37,38].

## Discussion

It has been a hot topic for researchers to determine the most effective surgical approach for THA elderly patients that has the most satisfying therapeutic effect with a low likelihood of postoperative problems.

In the present study, we conducted a meta-analysis to compare the DAA and the PLA approach to THA in elderly patients. Our analysis revealed that DAA reduced postoperative drainage, incision length, and blood loss and improved postoperative bed rest duration, length of stay in the hospital, and surgery duration. Since DAA is less invasive than PLA for THA in elderly patients with lower surgical stress, the results demonstrated a statistically significant difference in favour of DAA. In contrast, the meta-analyses by N. Ramadanov et al. [39] found similarly prolonged operation times in THA through DAA. DAA had a shorter incision length than CAs; DAA had similar results compared to CAs for blood loss. To further validate our conclusion, a sensitivity analysis was performed. The evidence quality of most of our analyses was low to middle.

Our meta-analysis targeted elderly patients and is comprehensive considering analyzing the following outcomes: HHS at 1 week, 1 month, 3 months, 6 months, and 12 months postoperatively; VAS at 3 days, 7 days, 1 month, 6 months, and 12 months postoperatively; incision length, duration of surgery, blood loss, length of hospital stay, postoperative drainage, duration of postoperative bed rest, acetabular abduction angle, and acetabular anteversion angle. To the best of our knowledge, this study involves the most comprehensive meta-analysis to evaluate DAA and PLA for THA in elderly patients.

HHS was used to assess the function of the hip joint, and our analysis results revealed significant differences between the two approaches in terms of HHS score at 1 month and 12 months postoperatively. The network meta-analysis conducted by Putananon et al. [8] conducted a network meta-analysis to compare DAA and PLA in THA and found that DAA was a preferable approach for improving postoperative VAS and HHS, but they only compared the VAS and HHS at the final follow-up. The meta-analysis by N. Ramadanov et al. [39] showed no difference in HHS 3, 6 and 12 months postoperatively in THA through DAA compared to THA through CAs. In our study, the VAS and HHS were classified at several time points postoperatively. According to our meta-analysis, DAA was better than PLA considering the HHS at 1 month and 12 months postoperatively, while no significant differences were observed between the two approaches regarding the HHS at 1 week, 3 months, and 6 months postoperatively. It was found that DAA was not related to the VAS score at 3 days, 7 days, 1 month, 6 months, and 12 months postoperatively in comparison with PLA. The overall results showed a tendency towards a better outcome in THA through DAA compared to THA through PLA. The improved functional outcome found in THA through DAA follows the lower tissue and muscle damage, presumably because of operating in a muscle-sparing anatomical plane. The improvement of HHS and relief of pain severity by DAA may be attributed to its muscle-sparing nature and less damage to soft tissue, compared with PLA [40].

In the present meta-analysis, these studies reported less mean hospitalization time, shorter operative time, and earlier duration of postoperative bed rest. Nevertheless, the PLA group generally required longer skin incision length and reported more blood loss and postoperative drainage during surgery. This indicates that PLA does more harm to the soft tissue than DAA, suggesting that the minimal invasion of DAA is attributed to less soft tissue injury and less amount of blood transfusion which is important to older patients.

The comparison of DAA and PLA for THA in terms of complications (postoperative dislocation, intraoperative fracture, lateral femoral cutaneous nerve (LFCN) neurapraxia, wound infection, and deep vein thrombosis) revealed that no significant differences emerged between the two approaches regarding intraoperative fracture, wound infection, and deep vein thrombosis (*p* > 0.05). However, DAA was found to be associated with a lower risk of postoperative dislocation, while the incidence of LFCN injury was higher in the DAA group than that in the PLA group with a significant difference. Only one study [38] involving 96 patients (48 in the DAA group and 48 in the PLA group) reported the rate of aseptic loosening at 2.08% in the PLA group.

The survival rate of artificial implants can be affected by the placement of femoral components [41]. The meta-analysis results revelated no significant differences in acetabular anteversion angle and abduction angle between the DAA and PLA groups [39]. What’s more, the mean cup inclination and anteversion angle of the DAA and PLA groups were almost within Lewinnek’s safe zone [42]. Therefore, we think that both DAA and PLA are effective for THA from the radiography perspective.

## Conclusion

Compared with PLA, DAA is related to earlier functional recovery and less invasion to help elderly THA patients return to normal activities. It was discovered that postoperative dislocation was uncommon for DAA. High-quality RCTs with large sample sizes that take account of complications, including lateral femoral cutaneous nerve injury and incidence of postoperative dislocation, during a long-term follow-up are required to verify these findings.

## Limitation

Our study has some limitations. First, more high-quality RCTs are needed to further investigate the effects of DAA on THA patients. Second, the inclusion of retrospective studies for meta-analysis inevitably resulted in selection bias. Third, differences in postoperative rehabilitation may lead to heterogeneity in the outcome. Four, we searched four English databases without limiting the language of the search. After screening strictly, irrelevant studies were excluded, and most of the included literature were from China. We respect the objective facts of the screened results. However, for the sake of the rigour and reliability of the results, we evaluated the quality of the literature strictly by the Cochrane bias risk assessment tool, and the results suggested that the overall literature quality was good. Although Chinese literature does not affect the results and conclusions, the results of a single language have certain limitations. We look forward to more studies from all over the world to verify and enrich our results in the future. Besides, the high risk of bias in participant blinding affects the accuracy of the results.

## Data Availability

Data sharing does not apply to this article as no datasets were generated or analyzed during the current study.
